# Personalized Nutrition with Banked Human Milk for Early Gut Microbiota Development: In Pursuit of the Perfect Match

**DOI:** 10.3390/nu16131976

**Published:** 2024-06-21

**Authors:** Emilia Hick, Marta Suárez, Alejandra Rey, Laura Mantecón, Nuria Fernández, Gonzalo Solís, Miguel Gueimonde, Silvia Arboleya

**Affiliations:** 1 Department of Microbiology and Biochemistry of Dairy Products, Instituto de Productos Lácteos de Asturias, Consejo Superior de Investigaciones Científicas (IPLA-CSIC), 33300 Villaviciosa, Spain; emilia.hick@ipla.csic.es (E.H.); a.rey.marino@ipla.csic.es (A.R.); 2 Pediatrics Service, Central University Hospital of Asturias (HUCA-SESPA), 33011 Oviedo, Spain; msr1070@hotmail.com (M.S.); laura_mantecon@hotmail.com (L.M.); solisgonzalo@uniovi.es (G.S.); 3 Instituto de Investigación Sanitaria del Principado de Asturias (ISPA), 33011 Oviedo, Spain; 4 Pediatrics Service, University Hospital of Cabueñes (CAB-SESPA), 33394 Gijón, Spain; nuriajmhd@gmail.com

**Keywords:** donated human milk, bank milk, breast milk, gut microbiota, early life, health, nutrition

## Abstract

The correct initial colonization and establishment of the gut microbiota during the early stages of life is a key step, with long-lasting consequences throughout the entire lifespan of the individual. This process is affected by several perinatal factors; among them, feeding mode is known to have a critical role. Breastfeeding is the optimal nutrition for neonates; however, it is not always possible, especially in cases of prematurity or early pathology. In such cases, most commonly babies are fed with infant formulas in spite of the official nutritional and health international organizations’ recommendation on the use of donated human milk through milk banks for these cases. However, donated human milk still does not totally match maternal milk in terms of infant growth and gut microbiota development. The present review summarizes the practices of milk banks and hospitals regarding donated human milk, its safety and quality, and the health outcomes in infants fed with donated human milk. Additionally, we explore different alternatives to customize pasteurized donated human milk with the aim of finding the perfect match between each baby and banked milk for promoting the establishment of a beneficial gut microbiota from the early stages of life.

## 1. Introduction

During the last decade, a growing body of knowledge has evidenced a compelling set of connections between the microbiota and human health [1]. The gut microbiota is the myriad of microorganisms living in the gastrointestinal tract of humans and animals, which have experienced co-evolution with them and perform important functions related to physiology, metabolism, immunity or protection [2]. The correct initial colonization and establishment of the gut microbiota during the early stages of life is a key step, with long-lasting consequences throughout the entire lifespan of the individual [3]. Disruptions in the early bacterial colonization during infancy can increase later vulnerability to various illnesses [4]. This process of early microbiota establishment is affected by several perinatal factors; among them, feeding mode is known to have a critical role [3].

Breast milk intake during infancy is crucial for optimal colonization and maturation of the infant microbiota. Milk compounds, such as oligosaccharides, promote the growth of dominant and beneficial bacteria, such as *Bifidobacterium*, *Bacteroides* or *Lactobacillus*, in human’s early life, in addition to the benefits of the microorganisms present in the milk [5,6]. Furthermore, breastfeeding has been associated with several beneficial physiological effects, including lower body mass index, reduced risk of obesity and diabetes later in life, decreased risk of atopic diseases, improvement in neurocognitive development, protection against pathogenic infections, etc. [7]. However, breastfeeding is not always possible, especially in cases of prematurity or early pathology. In such cases, most commonly babies are fed with infant formulas. This is in spite of the official nutritional and health international recommendation on the use of donated human milk (DHM) through human milk banks (HMBs) for these cases [8]. Therefore, DHM should be a preferable option to formula milk, but still recent studies indicate that DHM does not totally match maternal milk in terms of infant growth and development [9]. Moreover, the gut microbiota has been reported to differ between DHM and own-mother milk (OMM) fed infants [10,11]. These differences are not well understood yet but they could be due to milk processing at the milk banks (pasteurization, etc.), or even to differences in the ability of the infants’ microbiota to metabolize the components present in this foreign milk.

The establishment process of the gut microbiota is a key “window” period for manual intervention to establish a healthy microbiota [12] and personalizing the use of DHM could aid in the proper intestinal and physiological development of the baby. In this scenario, a major challenge ahead is not only to define in detail the modifications produced by milk processing in each component, but also to search for alternatives that promote the supplementation of DHM with the components which are lacking, according to the donor/recipient characteristics, to achieve optimal personalized feeding with DHM for each baby. Moreover, to be able to match a specific type of donated milk with an individual baby based on its microbiota capability to metabolize the different milk oligosaccharides would be a thrilling future challenge.

The present review summarizes the practices of HMBs and hospitals regarding DHM, its safety and quality, and the health outcomes in infants fed with donated milk. Additionally, we explore different alternatives to customize pasteurized DHM with the aim of finding the perfect match for each baby feeding with milk from HMBs and establishing a beneficial gut microbiota in the early stages of life.

## 2. Gut Microbiome Development: Early Health Foundation

The moment when colonization starts has been a matter of great scientific discussion. Colonization may begin in utero, as shown by the results of some studies in which culture and molecular techniques have been used to detect bacteria or bacterial DNA present in the intrauterine environment [13,14,15,16,17,18]. Although this hypothesis remains controversial [19,20,21], it seems that a maternal–fetal transmission of microbiota or microbial metabolites may occur during pregnancy and it may play a role in shaping postnatal development [22,23]. Moreover, prenatal factors may influence this initial colonization process. In primate models, it has been shown that a high-fat diet in the mother during pregnancy, independent of obesity, alters the microbiota of the offspring, observing a reduction in microbial diversity with effects that lasted up to one year of age, and persistently shapes the juvenile gut microbiome [24]. In a longitudinal human cohort, it was found that, similar to primates, a maternal high-fat diet shapes the neonatal gut microbiome, and that changes can persist through 4–6 weeks of age [18]. Infant fecal microbial composition was correlated with body weight and weight gain during pregnancy in a prospective follow-up study of mother–infant pairs [25]. Antibiotic treatment during pregnancy can also alter the composition of the newborn’s microbiota, which could lead to an alteration in its immune development [26,27]. A similar study reported that gestational diabetes mellitus altered the microbiota of both the mother and the newborn, resulting in a metabolic depletion in neonatal intestinal microbiota, and increased the prevalence of certain viruses in the meconium [28].

What is clear is that birth involves the greatest microbial challenge for the newborn and has a profound influence on the neonate’s gut microbiota. Vaginally born babies are in contact with their mother’s vaginal and fecal microbiota during labor, from which they will acquire an inoculum that colonizes the intestine, forming a microbiota composed mainly of *Lactobacillus*, *Prevotella*, *Atopobium*, *Bacteroides*, *Escherichia/Shigella* and *Bifidobacterium* genera members [29,30,31]. On the contrary, babies born by Cesarean section (C-section) are first exposed to the microbiota of the mother’s skin and bacteria from the hospital environment, so their gut microbiota will initially be colonized by *Staphylococcus* spp., *Klebsiella* spp. and *Escherichia coli* [32,33,34] and, show lower levels of *Bifidobacterium* and *Bacteroides* [30] and low bacterial richness and diversity [35]. Furthermore, vaginally born babies share more bacterial species with their mothers than C-section ones [36], mainly *Lactobacillus* during the first days of life followed by *Bacteroides* and *Bifidobacterium* in the second week [37]. These differences are significant during the first 6–12 months of life and some authors affirm that they disappear with age [33,38]; however, other authors reported differences even until 7 years of life between babies born by C-section and vaginally delivered, despite that they were less pronounced than early in life [39]. It has been suggested that the late microbial succession observed in these C-section infants could cause long-term effects on their immune and metabolic development [40,41]. Differences have also been observed in the microbiota of elective versus emergency C-section, as well as both vaginal and cesarean deliveries in which intrapartum antibiotics have been administered. Some of these changes persist up to 12 months of age, especially in formula-fed infants, such as increased Clostridiales and decreased *Bacteroidaceae* [42]. Gestational age is another factor that influences the gut microbiota composition, with a lower abundance of *Bifidobacterium* and Bacteroidetes and a higher abundance of *Enterobacteriaceae*, *Enterococcaceae* and *Lactobacillaceae* observed in preterm neonates compared to term neonates [43]. Necrotizing enterocolitis (NEC) is an inflammatory disease of the gastrointestinal tract that mainly affects premature infants [44] and has also been associated with a dysbiosis and delayed maturation of the gut microbiota [45,46,47].

After birth, the consecutive order in which bacteria colonize the intestine will influence the assembly of the microbial community and its ecological success [48]. During the first 6 months of age, infant’s gut microbiota evolves under the selective pressure of milk, which probably causes the decrease in diversity observed immediately after birth [49,50]. In the first days of life, facultative anaerobic bacteria predominate, such as streptococci and enterobacteria, which progressively create an anoxic environment in which strict anaerobic bacteria can proliferate, such as the bifidobacteria obtained from breast milk, which will be predominant within the first weeks of life [6]. Type of feeding influences the composition of the infant’s microbiota, with differences observed between breastfed and formula-fed infants. In the former, there are high levels of *Bifidobacterium*, including *B. breve*, *B. bifidum*, and *B. longum* [6,51,52,53], while in the latter an increase in *Enterobacteriaceae*, *Bacteroidaceae*, and *Clostridiaceae* is observed [6,35,54]. Breast milk provides a more balanced and stable intestinal microbiota in babies [55], which in turn contributes to better intestinal tolerance and an immunological protection against infections and other diseases. The cessation of breastfeeding represents another change in the infant’s microbiota [6,56], which transitions from a bifidobacteria-dominated microbiota to a more diverse [5,57]. Therefore, the infant gut microbiome undergoes a step-wise process of colonization, development and maturation, which is influenced by several factors, including both external [58] and host factors [59]. This process is defined by the dynamics of the different phyla in terms of abundance and changes in alpha-diversity [60]. In general terms, we can say that, in the early days, during lactation and until weaning, the diversity is low and the microbiota of the baby is dominated by bifidobacteria. Then, alpha-diversity and the detected phyla gradually change, and Bacteroidota and Pseudomonadota phyla diversify, with a predominance of Bacillota. Finally, when a family diet pattern is already set in infant habits at around 3 years of age, alpha-diversity and microbial composition at phylum level remain more or less stable [60].

Some other genetic and environmental factors also shape infant’s microbiota in the first stage of life, including the number of cohabitants, having older siblings, or the sex of the infant [61,62,63,64]. These variables facilitate the social transmission of bacteria. The administration of antibiotics early in life can affect the development of the microbiota, and these effects can be transitory or last over time [65]. Several studies show that antibiotic agents and other drugs increase the risk of developing immune-mediated diseases, such as cow milk protein allergy, diabetes, or asthma [66]. In a longitudinal study, Bunyavanich et al. found that gut microbiome composition at 3–6 months of age was associated with milk allergy resolution by age 8 years, with enrichment of Clostridia and Bacillota in these infants [67]. Antibiotics also increase the risk of being overweight later in life [40,68]; the younger, the more frequent this association, and the larger spectrum of antibiotic administered, the stronger the association [68]. The development of atopic disorders is also influenced by dysbiosis in early life gut microbiota, such as allergic rhinitis, asthma or atopic dermatitis [69,70,71]. In this sense, Penders et al. found that Clostridia colonization in 5–13 week old babies was associated with a higher risk of developing atopic dermatitis [72], whereas Ta et al. found that there was an enrichment of *Enterobacteriaceae* at three weeks of life and a delay in *Bactereroidaceae* colonization in children with this disease [73]. Differences between the gut microbiota of allergic and non-allergic infants and children are present before symptoms develop [74], pointing out the key role of the early establishment of the microbiota in the development of some diseases.

Early reports demonstrated that the microbiota undergoes most of its development very early in life and that the impression of these colonizers will have a short- and large-term impact. In this scenario, it is of vital importance to secure correct gut colonization and the early life nutrition will be crucial to this end. Being able to personalize the feeding in this early stage in those infants who are not breastfed, giving them donor milk with optimal characteristics for promoting an optimal gut microbiota development, will represent a great challenge and an opportunity in forthcoming years.

## 3. Early Life Nutrition: The Game Changer

Breastfeeding is considered the gold standard for infant nutrition, since it covers energy requirements and provides nutrients and bioactive compounds, which support optimal growth and promote life-long health [75]. Beyond its nutritional benefits, breast milk profoundly impacts on immune maturation, reducing gastrointestinal and respiratory infections in early life, as well as improving neurodevelopment, particularly in preterm babies [76]. Moreover, breastfeeding is known to decrease the risk of allergies, and non-communicable diseases during childhood [68]. In addition, lactation promotes good physical and psychological recovery of the mother after childbirth, improving infant care and reducing health costs for both individual families and the public socioeconomic burden [77,78]. That is why the World Health Organization (WHO) and the United Nations International Children’s Emergency Fund (UNICEF), in their 2014 policy report, strongly recommend human breast milk as the exclusive food from the first hour of life up to six months of age, combined with complementary solid foods thereafter, until two years of age or beyond [79].

Human milk contains all the macro- and micro-nutrients essential for newborns, in adequate amounts to enhance their growth and development. In fact, this biological fluid is considered a “living tissue” as it is composed of immune cells, microorganisms and a myriad of biologically active molecules, all of which have protective effects on the health of infants [78]. These nutritional and bioactive constituents vary among mothers, depending on genetic, lifestyle and perinatal factors. Milk composition also changes within the same woman, according to the stage of lactation, to meet the needs of the growing infant [80]. The most basic components are macro-nutrients: carbohydrates, proteins and fats, that provide 65–70 kcal of energy per 100 mL of mature milk. Lactose is the main carbohydrate, being present in a higher concentration in human milk that in that of other species. Fats are the second most prevalent macro-molecule in human milk, being essential for infant weight gain and development of the central nervous system. Protein compounds include a mixture of whey, caseins and peptides that supply essential amino acids, as well as bioactive proteins (e.g., enzymes and signaling peptides) that play an auxiliary role in digestion and utilization of other nutrients [81].

In addition to macronutrients, human milk has many non-nutritional bioactive components that profoundly impact infant health and growth. Mother’s own milk provides exposure-specific antibodies (sIgA, sIgG, sIgM), immunologic proteins (α-lactalbumin, lactoferrin, lysozyme) and cytokines (TNFα, IL-10, IFN-γ, TGF-β), which bring valuable compensation to the neonate’s immature immune system [82]. Another important and exclusive component of breastfeeding is that of human milk oligosaccharides (HMOs). HMOs are a group of more than 200 structurally complex and diverse unconjugated glycans, which represent the third most abundant solid component in breast milk, after lactose and lipids [83]. Moreover, as other body fluids, mother’s own milk contains exosomes that transport bioactive molecules. MicroRNA (miRNA) included in mother’s own milk-involves exosomes which deserve a special mention, due to their involvement in the development, differentiation, proliferation, metabolism, and death of cells and tissues. Each miRNA has been shown to regulate the expression of multiple genes [81].

Among the bioactive components previously named, HMOs play a special role in the establishment of a balanced infant gut microbiota [83]. While these oligosaccharides are not digestible by the host, they can be metabolized by certain commensal and beneficial bacteria (i.e., *Bifidobacterium* or *Bacteroides* species), stimulating their dominance and protecting against infection. HMOs also strengthen the epithelial barrier and support immune and cognitive function. Additionally, short-chain fatty acids, derived from HMO assimilation by gut associated bifidobacteria, represent an important energy source for enterocytes’ maturation [84]. The amount and distribution of HMOs vary greatly between mothers, being highly influenced by secretor status that determines the expression of FUT2 enzyme, leading to the synthesis of 2′-fucosyllactose (2′FL) in human milk [85].

**Figure 1:**
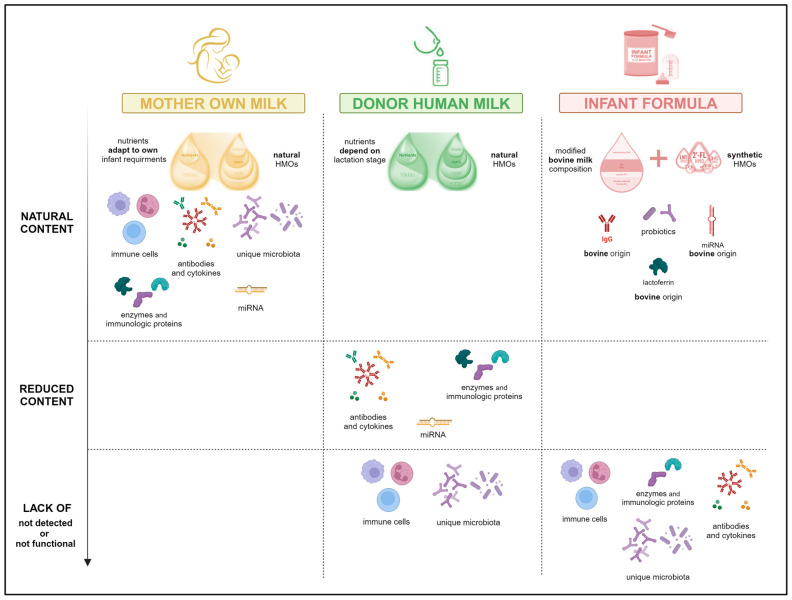
Comparison of the main nutritional and bioactive components between different infant´s feeding sources: mother’s own milk, pasteurized donor human milk and commercial infant formula.

On the other hand, infant formula, typically made from cow’s milk, is an option for babies who, for different reasons, cannot receive maternal milk. Although commercial formulas contain nutritional profiles adapted to the needs of the infant, these products do not naturally contain the bioactive and immunomodulatory components characteristic of breastfeeding. While some formulas are supplemented with particular compounds (i.e., miRNA, oligosaccharides, probiotics), their structures are not identical to those of human varieties and are affected by the manufacturing process. Moreover, formulas lack the dynamic capability of human milk, which adapts to the infant’s stages of growth and development [86,87] (Figure 1).

OMM is the preferred mode of infant feeding, especially in difficult situations, such as prematurely, to achieve optimal health and growth. However, there are circumstances which often make mothers of such babies unable to successfully breastfeed, requiring the implementation of alternative feeding modes. In such scenarios and thanks to the global increase in the network of HMBs, the WHO recommends that infants receive DHM instead of formula when breast milk is not available [8,88]. There is evidence that DHM improves feeding tolerance and provides protection against NEC, late-onset sepsis and other comorbidities in both preterm and term newborns [89]. Routine safety processing of DHM at the HMBs includes pasteurization, which eliminates the microbiota of breast milk and reduces the availability of several bioactive compounds [90]. However, other important components of human milk, such as HMOs, are resistant to heat treatment. In addition, donor milk respects the variation in milk composition throughout the stages of lactation, allowing optimal DHM to reach each recipient infant [91].

In the context of the immature metabolic and immune systems of the newborn, a crucial outcome of breastfeeding is the adequate colonization of the gastrointestinal tract [92]. The mode of infant feeding is a major determinant of early gut microbiota development and this critical process has been associated with lifelong gut microbiome composition and, consequently long-lasting physiological effects. Human milk carries its own unique microbiota and acts as a continuous source of health promoting bacteria for the infant gut [7]. In the new worldwide trend of personalized nutrition, the early stages of life offer great opportunities. Several efforts have been undertaken to advance the mimicry of human milk composition in the development of infant formulas, but the use of the DHM according to the neonate’s needs has not been explored yet and opens up a workable option with potential health benefits over the long term.

## 4. Bank Milks and Hospital Practices: Policy Regarding Donor Human Milk

DHM preserves some properties that are beneficial for babies, such as preterm infants, including faster gastric emptying, faster achievement of full enteral feeding, improved gut growth and maturation, decreased risk of NEC and late onset sepsis, and improved neurodevelopmental outcomes [93,94].

The growing recognition of the benefits of DHM has led to an increased global interest in establishing and maintaining HMBs to meet the need for donor milk. A milk bank is a service established to recruit breast milk donors, and collect, evaluate, store, process and distribute DHM [94,95]. Since the first HMBs were established in 1909 in Vienna (Austria), and a few years after at the Boston Floating Hospital (United States), many others have followed worldwide. In the 1960s, due to the development of high-quality infant formulas, the number of new HMBs decreased. In the 1980s, the first known cases of human immunodeficiency virus (HIV) occurred and, as it is transmissible through breast milk, many milk banks were closed. Since the early 2000s, proper screening of donating mothers, as well as adherence to procedural standards, has caused the number of HMBs to increase again [95]. Globally, there is a growing interest in milk banks worldwide. Additionally, there is currently a movement to open milk banks in low- and middle-income countries [93].

It has been questioned as to whether the benefits of DHM justify the expense, as HMBs charge a high processing fee (6–7 dollars/100 mL donor milk). However, it is inappropriate to pose this question because evidence has demonstrated that DHM saves lives. Several studies have analyzed the economic impact of human milk’s use, and most of them have documented that the use of DHM is cost-effective [95]. Johnson et al. demonstrated the cost-effectiveness of OMM supplemented with donor milk versus OMM supplemented with formula for very low birth weight infants admitted to the neonatal invasive care unit (NICU) [96]. In this study, the first group of patients had a lower incidence of NEC. They speculated that, with the use of DHM in those NICUs with high rates of NEC, higher cost savings could be achieved [96].

A human milk donor can be any healthy woman with a healthy lifestyle. Most of the milk is donated by women who have breastfed their own baby for some time and realize that their milk supply is large enough to allow them to donate milk while still meeting their own baby’s needs. The European Milk Bank Association (EMBA) makes some recommendations [97]:

- Donor screening should include an oral interview and completion of a health questionnaire.
- They will be required to undergo serological testing.
- Donors should inform the HMBs if there are any changes in their behavior or health status.
- Before accepting a donor’s milk, written informed consent for its use is obtained in accordance with the HMB protocols.
- Exclude donors if they smoke cigarettes; use recreational drugs; are known or found to be infected with HIV, hepatitis B or C, syphilis or human T-lymphotropic virus; use medications not on the EMBA approved medication list; have had a recent blood transfusion, tattoo, or piercing; follow a vegan diet without vitamin B12 supplementation; or have a sexual partner who has or is at risk of acquiring sexually transmitted infections.
- Train all new donors in handwashing and hygiene requirements for expressing, handling, storing, cooling, freezing, and transporting human milk.
- Provide appropriate ongoing support for all donors.

The most common recipients of donated human milk are [95]:

- Premature infants, especially those with a birth weight below 1500 g, due to their high risk of infection and NEC.
- Infants with gastrointestinal anomalies undergoing gastrointestinal surgery.
- Newborns at risk of intestinal ischemia.

It has been suggested that the availability of DHM could discourage mothers from providing milk to their own premature infants, but there is strong evidence that breastfeeding and donation complement each other and contribute to improving child health, through the exclusive feeding of all newborns [94,95]. Milk donation support practices are the most effective method of protecting, promoting and supporting breastfeeding. The presence of HMB in NICU represents a favorable element for supporting breastfeeding and significantly improves the availability of mother’s milk for feeding the premature baby and breastfeeding, with higher percentages of nutrition in mother’s milk at NICU discharge [94]. Mothers of premature infants often cannot provide milk at all or provide an insufficient amount of milk because premature delivery shortens the preparatory lactogenesis period. Additionally, the necessary mechanical milk expression is less effective in stimulating and maintaining milk production than suckling by a mature infant [95]. Moreover, the presence of HMBs results in the activation of standardized methods aimed at increasing breast milk production, providing lactation support from the NICU staff. A study carried out before and after opening the milk bank at the Hospital 12 de Octubre in Madrid (Spain) showed that the use of DHM reduced exposure to artificial formulas and increased the intake of OMM during hospital stay and the rate of exclusive breastfeeding at hospital discharge [98].

## 5. Donor Human Milk Processing: Safety and Quality

Milk delivered to HMBs must be pasteurized to ensure its microbial safety, inactivating viral and bacterial agents. The currently recommended processing, in all the international guidelines for the management of DHM in HMBs, includes Holder pasteurization (HoP), which consists in heating to 62.5 ± 1.5 °C for 30 min, followed by immediate freezing at −10 °C [99].

Pasteurized human milk is known to retain many beneficial components of fresh human milk. These heat-stable nutrients include lactose, fatty acids, the majority of minerals, and fat-soluble vitamins (A, D, and E) [100]. However, the thermal interventions negatively modify the raw milk, affecting some of its nutritional and biological properties [101]. Significant bioactive compounds, such as cytokines, hormones, growth factors, and immune cells, are decreased or inactivated by pasteurization. Additionally, this processing reduces the concentration of water-soluble vitamins and immunoglobulins and lowers the activity of signaling proteins and enzymes, such as lactoferrin, lysozyme, lipase, and amylase [100]. Moreover, HoP eliminates beneficial bacteria and antimicrobial peptides, thus resulting in the reduction of some bacteriostatic mechanisms, making the milk more susceptible to post-heating bacterial contamination [99]. In contrast, HMOs are resistant to pasteurization, remaining available in donated human milk [87]. After pasteurization, some of these compounds are replaced to recover the lost functionality, thus HMBs perform standard fortification based on quantitative analysis of macronutrients, and some milk banks pool milks from donors at different stages of lactation to homogenize macronutrient and fortification [102]. Other HMBs, in contrast, aim at a certain degree of personalization and classify DHM to match it to the recipient based on gestational age [91].

The optimization of the biological and nutritional quality of DHM is considered by the EMBA as a scientific and social priority. In order to investigate this aspect, a working group has been established, operating in different European countries, with the aim of evaluating. old and new methodologies in order to determine their effects on the quality of DHM [99].

Regarding recommendations for performing microbiological tests of donated milk before and after pasteurization, there is no consensus. The EMBA working group recommends that HMBs follow nationally or locally agreed guidance to adopt microbiological screening criteria [97]. One important aspect to consider when evaluating the processing of human milk is the viral inactivation effect of new methodologies. HoP has been shown to be capable of inactivating viruses transmitted through breast milk, such as HIV, human T-cell lymphotropic virus, and cytomegalovirus [99]. On the other hand, Igs conservation is often targeted as a qualitative/functional parameter in studies on alternative human milk pasteurization technologies [103].

Since heat treatments affect milk, different options have been explored and alternative technologies, such as high-temperature short-time pasteurization (HTST), high pressure processing (HPP), Ultraviolet-C irradiation (UV-C) and thermo-ultrasonication, have also being explored [99,103]. HTST is performed by heating thin layers of milk in continuous flow systems at 72 °C for 15 s and rapidly cooling it. It is a well-established heat treatment in the dairy industry [103]. This method is equivalent to HoP in ensuring microbiological safety of HM, but is better at preserving water soluble vitamins, lactoferrin and some cytokines [104]. HPP is a non-thermal processing method that involves applying high hydrostatic pressure (usually 400–800 MPa) through short-term treatments (<5–10 min). This alternative provides microbiologically safety, while better respecting the sensory and nutritional properties of human milk compared to HoP [103]. In particular, HPP has been found to be less damaging to exosomes and their miRNA content [100]. Irradiation is another non-thermal disinfection method that utilizes short-wavelength ultraviolet radiation (200–280 nm) in the UV-C region, which is harmful to microorganisms. Some reports have indicated that UV radiation can reduce the microbial load by 5 logarithmic orders and inactivate cytomegalovirus. UV-C treatment has also been shown to retain the bioactivity of lipase, lactoferrin and lysozyme, the fatty acid profile, and the levels of immunological proteins in fresh human milk [99]. Another alternative to HoP is ultrasonic processing (20–100 kHz) of HM. This technology induces inertial cavitation resulting in shock waves disrupting the cell membranes of the bacteria. Microbial inactivation rates can be improved by a combination with mild heating (45–50 °C); this treatment is called thermo-ultrasonication. Regarding the effect of ultrasonic treatment on enzymatic activity, lysozyme exhibited a retention of about 65% (lower after thermos-ultrasonication), and lipase was sensitive to both conditions, being retained at approximately 30% [103].

Currently, the EMBA recognizes that HoP is the safest compromise for the treatment of DHM; however, further studies are needed to improve this technology in order to minimize its effects on the biological components [99]. In addition, the knowledge of the specific components affected by the treatment would enhance the possibilities to tailor pasteurized human milk to the needs to each receptor baby. Regarding the delivery of pasteurized human milk to newborns, there are at least some consensus recommendations [97,105] (Table 1).

## 6. Microbiological and Health Outcomes in Infants: Good but Not Yet Equal

As previously stated, breastfeeding is considered the “gold standard” for infant nutrition for its high nutritional value, mainly attributed to HMOs [106]. Gut microbiota of breastfed infants have higher levels of *Bifidobacterium* compared to formula-fed infants [60,107,108]. The gut microbiota of full-term babies fed with human milk differs substantially from bovine-derived infant formula [109,110]. Several longitudinal studies showed that breastfed infants present a decrease in the levels of *Clostridium* over time, as well as an increase of *Bifidobacterium*, *Veillonella* and *Propionibacterium* [10,109,111], which can improve gut health by producing anti-inflammatory short chain fatty acids in these babies [112]. Besides, human milk mediates the interplay between the infant gut microbiome and immune system stimulation in early life and, when fed directly at the breast, a bi-directional parent-infant signaling appears to adapt it to the baby’s immunological needs, a process that is not observed when fed from a bottle [87]. Therefore, early life breastfeeding optimally regulates the development of the intestinal microbiota in infants, and health outcomes of this feeding type have been widely reported (Table 2). Human milk has been associated with a decreased risk of NEC in newborns, both term and preterm, compared to formula-fed infants [113,114,115,116], and it seems that human milk IgA plays a key role in preventing this disease [117]. Breastfeeding for at least the first 6 months of life prevents overweight and obesity in childhood [118,119,120], and it seems that this effect is dose-dependent [119,120,121], although some studies have not found this association [122,123]. Furthermore, it has been shown that formula feeding alters weight gain patterns [124,125], which could lead to rapid weight gain during childhood and a higher body mass index later [126]. Several studies show that exclusive breastfeeding in the first three to six months of life reduces the risk of developing atopic diseases during childhood, such as asthma [127,128] and atopic dermatitis [129] and extending the breastfeeding period (regardless of exclusivity) confers protection against these diseases even beyond age five [130].

On the other hand, formula-fed infants had increased richness of species with an overrepresentation of *Clostridioides difficile* [35,60,107,108,131,132]. However, supplementation with prebiotic agents of non-human origin, such as fructo-oligosaccharides (FOS) and galacto-oligosaccharides (GOS), can stimulate the development of *Bifidobacterium* and decreased *C. difficile* occurrence in the gut in comparison with unsupplemented formula-fed babies [133,134], even at the age of 12 months [135]. Although numerous efforts are being conducted to improve the composition of formula milk, this type of feeding is associated with an increased risk of developing NEC, respiratory infections, asthma, obesity, diabetes, and inflammatory bowel disease compared to exclusive breastfeeding [86,136]. Therefore, breastfeeding is the best choice for both full-term and preterm babies, in which breast milk has been shown to reduce the risk of sepsis [137], NEC [138] and retinopathy [77] in early life, but also the risk of suffering from cardiovascular disease, obesity and diabetes later in life [139]. It even seems that breast milk improves the neurocognitive development of preterm babies [140].

When the milk of the mother is not available, the use of DHM is the best alternative. However, donor milk does not seem to totally match OMM. The gut microbiota of preterm infants fed with DHM differ from those fed with OMM, showing different microbial profiles, such as a lower presence of *Bifidobacteriaceae* and a greater presence of *Staphylococcaceae*, *Clostridiaceae* and *Pasteurellaceae* [10]. In addition, in a study carried out on 42 premature babies, Arboleya et al. found a higher alpha-diversity of the bifidobacterial community in babies fed with DHM with respect to those fed with OMM [11]. Despite these differences, preterm infants fed with DHM showed a microbial profile closer to preterm fed with their OMM than to that formula fed. Furthermore, functional profiles between DHM and OMM preterm infants show no significant differences, revealing that it is the best feed type alternative [10]. However, the short- and long-term health effects of DHM feeding on premature babies needs to be evaluated. Knowing these differences in the composition of the intestinal microbiota in children breastfed with their OMM or DHM will help establish specific objectives to design personalized intervention strategies that contribute to the establishment of an adequate intestinal microbiota in the newborn.

## 7. Personalization of Pasteurized Human Donor Milk: The Next Opportunity

As previously indicated, all international guidelines for the management of HMBs recommend HoP to ensure the microbial safety of DHM. Pasteurization kills 99% of bacteria, including the unique maternal milk microbiome, and inactivates a large proportion of its bioactive components. In this context, supplementing donated milk with different sources of beneficial bacteria and/or prebiotics could mimic the advantages of breastfeeding with OMM [105,141]. Transfer of the mother’s own milk into the pasteurized DHM is an interesting option for recolonizing the milk with maternal microbiota, especially among mothers of premature and low birth weight infants. These mothers often produce insufficient volumes of milk to meet the needs of their newborn, but enough to enrich the donated milk received by their own baby. Studies have attempted this by inoculating small volumes (10 to 30% *v*/*v*) of mother’s own milk. After 4 h of incubation at 37 °C, the results have shown a reasonable restoration of specific microbes from the mother’s own milk, based on viable bacterial counts and microbiome analysis [142,143,144]. In addition to beneficial bacteria, transfer of the OMM can provide prebiotic compounds and a variety of antimicrobial proteins, which contribute to modulating early microbial colonization [141]. Another option is the enrichment of DHM with probiotics isolated from mother´s own milk. Findings indicate the transmission of health-promoting bacteria from mothers to their babies through breastfeeding. Human milk has been shown to be a good source of probiotics because these bacteria would meet some of the main criteria generally recommended: human origin, adaptation to dairy substrates, and a history of safe intake by babies [7,145]. In fact, several strains of potential probiotics have been isolated from breast milk, generally corresponding to species of *Bifidobacterium* and *Lactobacillus*. The European Food Safety Authority (EFSA) granted these the Qualified Presumption of Safety (QPS) status [146]. In this context, the generation of a collection of positive and safe strains isolated from OMM, and their addition to pasteurized DHM, would allow the restoration of a “synthetic milk microbiota” that contributes to the proper early intestinal colonization of babies.

Compared to OMM transfer, supplementation with specific probiotics ensures the supply of proven beneficial bacteria, avoiding the possibility of exposure to potential pathogens harbored in the breast milk. In contrast, receiving a wide range of maternal microbes could be advantageous for early immune programming, more similar to exclusive feeding with mother´s own milk. Additionally, it is important to note that, besides bacteria, HoP eliminates immune cells and proteins, which play a key regulatory role in modulating the infant’s gut microbiota. Therefore, the inclusion of bacteria in HoP DHM, which lacks these important immune regulators, could pose a risk to vulnerable infants. Transplantation of the global mother´s own milk microbiome, involving the administration of bacteria along with immune bio-actives and microbiota modifiers, would appear to be a better strategy for enriching DHM [141]. However, the feasibility and safety of these strategies for restoring the microbiota of DHM should be reinforced by further studies.

On the other hand, as mentioned before, HMOs are the main prebiotic component of human milk. These oligosaccharides act as selective nutrients for beneficial bacteria in the infant’s gut microbiota. Although HMOs are resistant to routine HoP of DHM, recent data have revealed that not all infants benefit equally from these oligosaccharides. Some gut microbiotas are better at fermenting specific HMOs, stimulating the growth of beneficial species and secreting immune and metabolic regulatory compounds [85,147]. Such microbiotas are called “fast degraders” [148,149] and are usually dominated by certain species of *Bifidobacterium (B. breve*, *B. longum* subsp. *infantis*, and *B. bifidum)* and *Bacteroides (B. fragilis*, *B. vulgatus*, and *B. thetaiotaomicron*) [149,150]. The presence of a fast or slow degrader microbiota could be associated with genetic and phenotypic characteristics (secretor status) of both the mother and the baby, which determine the composition of breast milk, its influence on intestinal colonization, and the response of this early microbiota to HMOs. These findings entail a new challenge in this research field, which is to ensure the “perfect match” between the DHM selected from the HMB and the recipient baby on the basis of the metabolic capabilities of the baby’s microbiota. Understanding the key factors determining the ability of the infant microbiota to metabolize the different HMOs present in different DHM may allow the development of fast screening procedures for the personalization of milk use at the HMBs.

Each mother has a unique milk microbiome that can be partially restored with different novel approaches to enhance the bioactivity of DHM and tailor it to each recipient baby. Several findings indicate that the OMM microbiome, as part of milk composition, is influenced by various factors (e.g., gestational age and maternal diet) and varies throughout lactation stages. Moreover, it changes within the same feeding session, likely due to the transfer of oral bacteria from the baby during breastfeeding, suggesting that milk-associated bacteria and HMOs guide the formation of the baby’s gut microbiota [151]. In this regard, DHM from mothers who have delivered prematurely has the most suitable composition for preterm recipients, and the same consideration applies to term babies [93]. Personalizing the donated milk–baby dyad may contribute to a more robust establishment of the intestinal microbiome, mimicking the composition of the gut microbiota of breastfed infants and allowing the baby to maximize the potential of breast milk. Consequently, this tailored nutrition will provide both short- and long-term health benefits.

## 8. Conclusions, Perspectives and Future Trends

It is widely accepted that the infant gut microbiota plays a crucial role in infant health and that a correct establishment early in life will entail long-lasting effect throughout life. While breast milk is the gold standard for neonates and infants, it is not always available. DHM is then the best alternative, and there are there are increasingly more hospitals hosting milk banks for this practice. Donated milk undergoes a thermal process to ensure its safety, but some bioactive components are affected and trying to restore them constitutes a challenging task.

In this review, we have summarized the early gut microbiota establishment and the different factors affecting this process as the backbone for future health, underscoring the early life nutrition as a key player. We have shed light on milk bank and hospital practices in DHM processing, and we have further emphasized the advantages of DHM as the best option for infants who cannot receive their OMM. We provided an overview of the current knowledge on the differences in both the composition of DHM before and after processing, and the gut microbiota composition of babies fed by both alternatives (DHM and OMM). We have tried to underline that the biological basis for these differences should set the targets for optimizing the use of donor human milk. On the one hand, the addition of missing components could enhance the benefits of DHM, using, for example, probiotics, prebiotics or even HMOs. On the other hand, this review has addressed an upcoming challenge: the customization of donated milk for each individual baby. Knowledge about the capability of each infant´s gut microbiota to metabolize the different components of breast milk would allow the selection of the best DHM at HMBs for each individual infant, “the perfect match”. These alternatives would allow the customization and optimization of DHM use, closing the gap regarding the health effects of OMM for the baby and maximizing the benefits of this practice.

## Figures and Tables

**Table 1:** Recommendations for the delivery of pasteurized donated human milk.

Items	Recommendations
Labeled	All donated human milk and containers should be labeled at each stage to ensure traceability and tracking of the milk.
Thawing	Donated human milk containers should be thawed in a refrigerator (4 °C) for 24 h. In urgent and exceptional cases, containers can be thawed at room temperature. A microwave oven has never to be used to thaw or heat the milk. Thawed donated human milk should be used within 24 h and should not be refrozen.
Administration	Donated human milk can be administered using a cup, spoon, or through small tubes that the baby sucks along with a pacifier, or through gastric tubes according to clinical guidelines.
Volumes	The required volume of donated human milk is based on the newborn’s age, gestational age and weight, as well as feeding tolerance. This volume represents the difference between recommended intake and the quantity of mother´s own milk available.
Consent	Before administration of donated human milk, informed consent is required from recipient´s parents.
Duration	Decisions about the need to continue with donated human milk are regularly reviewed, taking into account the baby’s growth and nutritional requirements. The hospital should record how donated milk is used, including in the baby’s hospital notes.

**Table 2:** Type of infant feeding, characteristics of gut microbiota, and health outcomes.

Type of Feeding	Characteristics of Gut Microbiota	Health Outcomes
Breastfeeding	*↑ Bifidobacterium*, *Veillonella*, *Propionibacterium*	Breastfeeding
Formula	*↑ Clostridioides difficile*	Increased risk of NEC, respiratory infections, asthma, obesity, diabetes, and inflammatory bowel disease
Donor human milk	↓* Bifidobacterium* ↑* Staphylococcaceae*, *Clostridiaceae* and *Pasteurellaceae* ↑ Alpha-diversity in bifidobacterial species	Short- and long-term effects not yet evaluated

↑ increment; ↓ decrement.

## References

[1] Fan Y., Pedersen O. (2021). Gut microbiota in human metabolic health and disease. Nat. Rev. Microbiol..

[2] Thursby E., Juge N. (2017). Introduction to the human gut microbiota. Biochem. J..

[3] Milani C., Duranti S., Bottacini F., Casey E., Turroni F., Mahony J., Belzer C., Delgado Palacio S., Arboleya Montes S., Mancabelli L. (2017). The First Microbial Colonizers of the Human Gut: Composition, Activities, and Health Implications of the Infant Gut Microbiota. Microbiol. Mol. Biol. Rev..

[4] Park H., Park N.Y., Koh A. (2023). Scarring the early-life microbiome: Its potential life-long effects on human health and diseases. BMB Rep..

[5] Yatsunenko T., Rey F.E., Manary M.J., Trehan I., Dominguez-Bello M.G., Contreras M., Magris M., Hidalgo G., Baldassano R.N., Anokhin A.P. (2012). Human gut microbiome viewed across age and geography. Nature.

[6] Bäckhed F., Roswall J., Peng Y., Feng Q., Jia H., Kovatcheva-Datchary P., Li Y., Xia Y., Xie H., Zhong H. (2015). Dynamics and Stabilization of the Human Gut Microbiome during the First Year of Life. Cell Host Microbe.

[7] Lyons K.E., Ryan C.A., Dempsey E.M., Ross R.P., Stanton C. (2020). Breast Milk, a Source of Beneficial Microbes and Associated Benefits for Infant Health. Nutrients.

[8] World Health Organization (2011). Guidelines on Optimal Feeding of Low Birthweight Infants in Low and Middle-Income Countries.

[9] Hård A.L., Nilsson A.K., Lund A.M., Hansen-Pupp I., Smith L.E.H., Hellström A. (2019). Review shows that donor milk does not promote the growth and development of preterm infants as well as maternal milk. Acta Paediatr..

[10] Parra-Llorca A., Gormaz M., Alcántara C., Cernada M., Nuñez-Ramiro A., Vento M., Collado M.C. (2018). Preterm gut microbiome depending on feeding type: Significance of donor human milk. Front. Microbiol..

[11] Arboleya S., Saturio S., Suárez M., Fernández N., Mancabelli L., de Los Reyes-Gavilán C.G., Ventura M., Solís G., Gueimonde M. (2020). Donated human milk as a determinant factor for the gut bifidobacterial ecology in premature babies. Microorganisms.

[12] Koenig J.E., Spor A., Scalfone N., Fricker A.D., Stombaugh J., Knight R., Angenent L.T., Ley R.E. (2011). Succession of microbial consortia in the developing infant gut microbiome. Proc. Natl. Acad. Sci. USA.

[13] Aagaard K., Ma J., Antony K.M., Ganu R., Petrosino J., Versalovic J. (2014). The placenta harbors a unique microbiome. Sci. Transl. Med..

[14] Gosalbes M.J., Llop S., Vallès Y., Moya A., Ballester F., Francino M.P. (2013). Meconium microbiota types dominated by lactic acid or enteric bacteria are differentially associated with maternal eczema and respiratory problems in infants. Clin. Exp. Allergy.

[15] Jiménez E., Fernández L., Marín M.L., Martín R., Odriozola J.M., Nueno-Palop C., Narbad A., Olivares M., Xaus J., Rodríguez J.M. (2005). Isolation of commensal bacteria from umbilical cord blood of healthy neonates born by cesarean section. Curr. Microbiol..

[16] Steel J.H., Malatos S., Kennea N., Edwards A.D., Miles L., Duggan P., Reynolds P.R., Feldman R.G., Sullivan M.H. (2005). Bacteria and inflammatory cells in fetal membranes do not always cause preterm labor. Pediatr. Res..

[17] Perez-Muñoz M.E., Arrieta M.C., Ramer-Tait A.E., Walter J. (2017). Critical assessment of the “sterile womb” and “in utero” colonization hypothesis: Implications for research on the pioneer infant microbiome. Microbiome.

[18] Collado M.C., Rautava S., Aakko J., Isolauri E., Salminen S. (2016). Human gut colonisation may be initiated in utero by distinct microbial communities in the placenta and amniotic fluid. Sci. Rep..

[19] Segata N. (2019). No bacteria found in healthy placentas. Nature.

[20] Bushman F.D. (2019). De-Discovery of the Placenta Microbiome. Am. J. Obstet. Gynecol..

[21] Walter J., Hornef M.W. (2021). A philosophical perspective on the prenatal in utero microbiome debate. Microbiome.

[22] Chu D.M., Meyer K.M., Prince K.L., Aagaard K.M. (2016). Impact of maternal nutrition in pregnancy and lactation on offspring gut microbial composition and function. Gut Microbes.

[23] Fujimura K.E., Slusher N.A., Cabana M.D., Lynch S.V. (2010). Role of the gut microbiota in defining human health. Expert Rev. Anti Infect. Ther..

[24] Ma J., Prince A.L., Bader D., Hu M., Ganu R., Baquero K., Blundell P., Alan Harris R., Frias A.E., Grove K.L. (2014). High-fat maternal diet during pregnancy persistently alters the offspring microbiome in a primate model. Nat. Commun..

[25] Collado M.C., Isolauri E., Laitinen K., Salminen S. (2010). Effect of mother’s weight on infant’s microbiota acquisition, composition, and activity during early infancy: A prospective follow-up study initiated in early pregnancy. Am. J. Clin. Nutr..

[26] Gonzalez-Perez G., Hicks A.L., Tekieli T.M., Radens C.M., Williams B.L., Lamousé-Smith E.S. (2016). Maternal antibiotic treatment impacts development of the neonatal intestinal microbiome and antiviral immunity. J. Immunol..

[27] Nyangahu D.D., Lennard K.S., Brown B.P., Darby M.G., Wendoh J.M., Havyarimana E., Smith P., Butcher J., Stintzi A., Mulder N. (2018). Disruption of maternal gut microbiota during gestation alters offspring microbiota and immunity. Microbiome.

[28] Wang J., Zheng J., Shi W., Du N., Xu X., Zhang Y., Ji P., Zhang F., Jia Z., Wang Y. (2018). Dysbiosis in maternal and neonatal microbiota associated with gestational diabetes mellitus. Gut.

[29] Dominguez-Bello M.G., De Jesus-Laboy K.M., Shen N., Cox L.M., Amir A., Gonzalez A., Bokulich N.A., Song S.J., Hoashi M., Rivera-Vinas J.I. (2016). Partial restoration of the microbiota of cesarean-born infants via vaginal microbial transfer. Nat. Med..

[30] Rutayisire E., Huang K., Liu Y., Tao F. (2016). The mode of delivery affects the diversity and colonization pattern of the gut microbiota during the first year of infants’ life: A systematic review. BMC Gastroenterol..

[31] Kumbhare S.V., Patangia D.V., Patil R.H., Shouche Y.S., Patil N.P. (2019). Factors influencing the gut microbiome in children: From infancy to childhood. J. Biosci..

[32] Reyman M., van Houten M.A., van Baarle D., Bosch A.A.T.M., Man W.H., Chu M.L.J.N., Arp K., Watson R.L., Sanders E.A.M., Fuentes S. (2019). Impact of delivery mode-associated gut microbiota dynamics on health in the first year of life. Nat. Commun..

[33] Hesla H.M., Stenius F., Jäderlund L., Nelson R., Engstrand L., Alm J., Dicksved J. (2014). Impact of lifestyle on the gut microbiota of healthy infants and their mothers- the ALADDIN birth cohort. FEMS Microbiol. Ecol..

[34] Shao Y., Forster S.C., Tsaliki E., Vervier K., Strang A., Simpson N., Kumar N., Stares M.D., Rodger A., Brocklehurst P. (2019). Stunted microbiota and opportunistic pathogen colonization in caesarean-section birth. Nature.

[35] Azad M., Konya T., Maughan H., Guttman D.S., Field C.J., Chari R.S., Sears M.R., Becker A.B., Scott J.A., Kozyrskyj A.L. (2013). Gut microbiota of healthy Canadian infants: Profiles by mode of delivery and infant diet at 4 months. CMAJ.

[36] Wampach M., Heintz-Buschart A., Fritz J.V., Ramiro-Garcia J., Habier J., Herold M., Narayanasamy S., Kaysen A., Hogan A.H., Bindl L. (2018). Birth mode is associated with earliest strain-conferred gut microbiome functions and immunostimulatory potential. Nat. Commun..

[37] Dominguez-Bello M.G., Costello E.K., Contreras M., Magris M., Hidalgo G., Fierer N., Knight R. (2010). Delivery mode shapes the acquisition and structure of the initial microbiota across multiple body habitats in newborns. Proc. Natl. Acad. Sci. USA.

[38] Hill C.J., Lynch D.B., Murphy K., Ulaszewska M., Jeffery I.B., O’Shea C.A., Watkins C., Dempsey E., Mattivi F., Tuohy K. (2017). Evolution of gut microbiota composition from birth to 24 weeks in the INFANTMET Cohort. Microbiome.

[39] Salminen S., Gibson G.R., McCartney A.L., Isolauri E. (2004). Influence of mode of delivery on gut microbiota composition in seven yearold children. Gut.

[40] Korpela K., Zijlmans M.A., Kuitunen M., Kukkonen K., Savilahti E., Salonen A., de Weerth C., de Vos W.M. (2017). Childhood BMI in relation to microbiota in infancy and lifetime antibiotic use. Microbiome.

[41] Gensollen T., Iyer S.S., Kasper D.L., Blumberg R.S. (2016). How colonization by microbiota in early life shapes the immune system. Science.

[42] Azad M.B., Konya T., Persaud R.R., Guttman D.S., Chari R.S., Field C.J., Sears M.R., Mandhane P.J., Turvey S.E., Subbarao P. (2016). Impact of maternal intrapartum antibiotics, method of birth and breastfeeding on gut microbiota during the first year of life: A prospective cohort study. BJOG.

[43] Arboleya S., Binetti A., Salazar N., Fernández N., Solís G., Hernández-Barranco A., Margolles A., de Los Reyes-Gavilán C.G., Gueimonde M. (2012). Establishment and development of intestinal microbiota in preterm neonates. FEMS Micobiol. Ecol..

[44] Hackam D.J. (2021). Necrotizing Enterocolitis: Pathogenesis, Diagnosis and Treatment.

[45] Claud E.C., Keegan K.P., Brulc J.M., Lu L., Bartels D., Glass E., Chang E.B., Meyer F., Antonopoulos D.A. (2013). Bacterial community structure and functional contributions to emergence of health or necrotizing enterocolitis in preterm infants. Microbiome.

[46] Zhou Y., Shan G., Sodergren E., Weinstock G., Walker W.A., Gregory K.E. (2015). Longitudinal analysis of the premature infant intestinal microbiome prior to necrotizing enterocolitis: A case-control study. PLoS ONE.

[47] Mai V., Torrazza R.M., Ukhanova M., Wang X., Sun Y., Li N., Shuster J., Sharma R., Hudak M.L., Neu J. (2013). Distortions in development of intestinal microbiota associated with late onset sepsis in preterm infants. PLoS ONE.

[48] Martinez M., Maldonado-Gomez M.X., Gomes-Neto J.C., Kittana H., Ding H., Schmaltz R., Joglekar P., Cardona R.J., Marsteller N.L., Kembel S.W. (2018). Experimental evaluation of the importance of colonization history in early-life gut microbiota assembly. eLife.

[49] Pantoja-Feliciano I.G., Clemente J.C., Costello E.K., Perez M.E., Blaser M.J., Knight R., Dominguez-Bello M.G. (2013). Biphasic assembly of the murine intestinal microbiota during early development. ISME J..

[50] Bokulich N.A., Chung J., Battaglia T., Henderson N., Jay M., Li H., Lieber A., Wu F., Perez-Perez G.I., Chen Y. (2016). Antibiotics, birth mode, and diet shape microbiome maturation during early life. Sci. Transl. Med..

[51] Laursen M.F., Andersen L.B., Michaelsen K.F., Mølgaard C., Trolle E., Bahl M.I., Licht T.R. (2016). Infant gut microbiota development is driven by transition to family foods independent of maternal obesity. Msphere.

[52] Palmer C., Bik E.M., DiGiulio D.B., Relman D.A., Brown P.O. (2007). Development of the human infant intestinal microbiota. PLoS Biol..

[53] Saturio S., Nogacka A.M., Suárez M., Fernández N., Mantecón L., Mancabelli L., Milani C., Ventura M., de Los Reyes-Gavilán C.G., Solís G. (2021). Early-Life Development of the Bifidobacterial Community in the Infant Gut. Int. J. Mol. Sci..

[54] Adlerberth I., Wold A. (2009). Establishment of the gut microbiota in Western infants. Acta Paediatr..

[55] Thompson A.L., Monteagudo-Mera A., Cadenas M.B., Lampl M.L., Azcarate-Peril M.A. (2015). Milk- and solid-feeding practices and daycare attendance are associated with differences in bacterial diversity, predominant communities, and metabolic and immune function of the infant gut microbiome. Front. Cell. Infect. Microbiol..

[56] Bergström A., Skov T.H., Bahl M.I., Roager H.M., Christensen L.B., Ejlerskov K.T., Mølgaard C., Michaelsen K.F., Licht T.R. (2014). Establishment of intestinal microbiota during early life: A longitudinal, explorative study of a large cohort of Danish infants. Appl. Environ. Microbiol..

[57] Gómez-Martín M., Saturio S., Arboleya S., Herrero-Morín D., Calzón M., López T., González S., Gueimonde M. (2022). Association between diet and fecal microbiota along the first year of life. Food Res. Int..

[58] Korpela K., de Vos W.M. (2018). Early life colonization of the human gut: Microbes matter everywhere. Curr. Opin. Microbiol..

[59] Planer J.D., Peng Y., Kau A.L., Blanton L.V., Ndao I.M., Tarr P.I., Warner B.B., Gordon J.I. (2016). Development of the gut microbiota and mucosal IgA responses in twins and gnotobiotic mice. Nature.

[60] Stewart C.J., Ajami N.J., O’Brien J.L., Hutchinson D.S., Smith D.P., Wong M.C., Ross M.C., Lloyd R.E., Doddapaneni H., Metcalf G.A. (2018). Temporal development of the gut microbiome in early childhood from the TEDDY study. Nature.

[61] Penders J., Thijs C., Vink C., Stelma F.F., Snijders B., Kummeling I., van den Brandt P.A., Stobberingh E.E. (2006). Factors influencing the composition of the intestinal microbiota in early infancy. Pediatrics.

[62] Penders J., Gerhold K., Thijs C., Zimmerman K., Wahn U., Lau S., Hamelmann E. (2014). New insights into the hygiene hypothesis in allergic diseases: Mediation of sibling and birth mode effects by the gut microbiota. Gut Microbes.

[63] Martin R., Makino H., Cetinyurek Yavuz A., Ben-Amor K., Roelofs M., Ishikawa E., Kubota H., Swinkels S., Sakai T., Oishi K. (2016). Early-life events, include mode of delivery and type of feeding, siblings and gender, shape the developing gut microbiota. PLoS ONE.

[64] Lane A.A., McGuire M.K., McGuire M.A., Williams J.E., Lackey K.A., Hagen E.H., Kaul A., Gindola D., Gebeyehu D., Flores K.E. (2019). Household composition and the infant fecal microbiome: The INSPIRE study. Am. J. Phys. Anthropol..

[65] Zeissig S., Blumberg R.S. (2014). Life at the beginning: Perturbation of the microbiota by antibiotics in early life and its role in health and disease. Nat. Immunol..

[66] Francino M.P. (2016). Antibiotics and the human microbiome: Dysbioses and accumulation of resistances. Front. Microbiol..

[67] Bunyavanich S., Shen N., Grishin A., Wood R., Burks W., Dawson P., Jones S.M., Leung D.Y.M., Sampson H., Sicherer S. (2016). Early-life gut microbiome composition and milk allergy resolution. J. Allergy Clin. Immunol..

[68] Miller S.A., Wu R.K.S., Oremus M. (2018). The association between antibiotic use in infancy and childhood overweight or obesity: A systematic review and metaanalysis. Obes. Rev..

[69] Abrahamsson T.R., Jakobsson H.E., Andersson A.F., Björkstén B., Engstrand L., Jenmalm M.C. (2012). Low diversity of the gut microbiota in infants with atopic eczema. J. Allergy Clin. Immunol..

[70] Abrahamsson T.R., Jakobsson H.E., Andersson A.F., Björkstén B., Engstrand L., Jenmalm M.C. (2014). Low gut microbiota diversity in early infancy precedes asthma at school age. Clin. Exp. Allergy.

[71] Bisgaard H., Li N., Bonnelykke K., Chawes B.L., Skov T., Paludan-Müller G., Stokholm J., Smith B., Krogfelt K.A. (2011). Reduced diversity of the intestinal microbiota during infancy is associated with increased risk of allergic disease at school age. J. Allergy Clin. Immunol..

[72] Penders J., Gerhold K., Stobberingh E.E., Thijs C., Zimmermann K., Lau S., Hamelmann E. (2013). Establishment of the intestinal microbiota and its role for atopic dermatitis in early childhood. J. Allergy Clin. Immunol..

[73] Ta L.D.H., Chan J.C.Y., Yap G.C., Purbojati R.W., Drautz-Moses D.I., Koh Y.M., Tay C.J.X., Huang C.H., Kioh D.Y.Q., Woon J.Y. (2020). A compromised developmental trajectory of the infant gut microbiome and metabolome in atopic eczema. Gut Microbes.

[74] Kalliomäki M., Kirjavainen P., Eerola E., Kero P., Salminen S., Isolauri E. (2001). Distinct patterns of neonatal gut microflora in infants in whom atopy was and was not developing. Distinct patterns of neonatal gut microflora in infants in whom atopy was and was not developing. J. Allergy Clin. Immunol..

[75] Walker A. (2010). Breast milk as the gold standard for protective nutrients. J. Pediatr..

[76] Hermansson H., Kumar H., Collado M.C., Salminen S., Isolauri E., Rautava S. (2019). Breast Milk Microbiota Is Shaped by Mode of Delivery and Intrapartum Antibiotic Exposure. Front. Nutr..

[77] Miller J., Tonkin E., Damarell R.A., McPhee A.J., Suganuma M., Suganuma H., Middleton P.F., Makrides M., Collins C.T. (2018). A Systematic Review and Meta-Analysis of Human Milk Feeding and Morbidity in Very Low Birth Weight Infants. Nutrients.

[78] García-Ricobaraza M., García-Santos J.A., Escudero-Marín M., Diéguez E., Cerdó T., Campoy C. (2021). Short- and Long-Term Implications of Human Milk Microbiota on Maternal and Child Health. Int. J. Mol. Sci..

[79] WHO/UNICEF (2014). Global Nutrition Targets 2025: Breastfeeding Policy Brief (WHO/NMH/NHD/14.7).

[80] Demmelmair H., Jiménez E., Collado M.C., Salminen S., McGuire M.K. (2020). Maternal and Perinatal Factors Associated with the Human Milk Microbiome. Curr. Dev. Nutr..

[81] Yi D.Y., Kim S.Y. (2021). Human Breast Milk Composition and Function in Human Health: From Nutritional Components to Microbiome and MicroRNAs. Nutrients.

[82] Demers-Mathieu V., Huston R.K., Markell A.M., McCulley E.A., Martin R.L., Spooner M., Dallas D.C. (2019). Differences in Maternal Immunoglobulins within Mother’s Own Breast Milk and Donor Breast Milk and across Digestion in Preterm Infants. Nutrients.

[83] Bode L. (2020). Human Milk Oligosaccharides: Structure and Functions. Nestle Nutr. Inst. Workshop Ser..

[84] Masi A.C., Stewart C.J. (2022). Untangling human milk oligosaccharides and infant gut microbiome. iScience.

[85] Dinleyici M., Barbieur J., Dinleyici E.C., Vandenplas Y. (2023). Functional effects of human milk oligosaccharides (HMOs). Gut Microbes.

[86] Almeida C.C., Mendonça Pereira B.F., Leandro K.C., Costa M.P., Spisso B.F., Conte-Junior C.A. (2021). Bioactive compounds in infant formula and their effects on infant nutrition and health: A systematic literature review. Int. J. Food Sci..

[87] Ames S.R., Lotoski L.C., Azad M.B. (2023). Comparing early life nutritional sources and human milk feeding practices: Personalized and dynamic nutrition supports infant gut microbiome development and immune system maturation. Gut Microbes.

[88] Parker M.G., Stellwagen L.M., Noble L., Kim J.H., Poindexter B.B., Puopolo K.M. (2021). Section on breatfeeding, Committee on nutrition, committee on fetus and newborn. Promoting human milk and breastfeeding for the very low birth weight infant. Pediatrics.

[89] Kashyap V., Choudhari S.G. (2024). Unlocking the Potential: A Systematic Literature Review on the Impact of Donor Human Milk on Infant Health Outcomes. Cureus.

[90] Gutierrez dos Santos B., Perrin M.T. (2022). What is known about human milk bank donors around the world: A systematic scoping review. Public Health Nutr..

[91] Sánchez Luna M., Martin S.C., Gómez de Orgaz C.S. (2021). Human milk bank and personalized nutrition in the NICU: A narrative review. Eur. J. Pediatr..

[92] Kapourchali F.R., Cresci G.A.M. (2020). Early-Life Gut Microbiome—The Importance of Maternal and Infant Factors in Its Establishment. Nutr. Clin. Pract..

[93] Tyebally Fang M., Grummer-Strawn L., Maryuningsih Y., Biller-Andorno N. (2021). Human milk banks: A need for further evidence and guidance. Lancet Glob. Health.

[94] Quitadamo P.A., Palumbo G., Cianti L., Lurdo P., Gentile M.A., Villani A. (2021). The Revolution of Breast Milk: The Multiple Role of Human Milk Banking between Evidence and Experience—A Narrative Review. Int. J. Pediatr..

[95] Haiden N., Ziegler E.E. (2016). Human Milk Banking. Ann. Nutr. Metab..

[96] Johnson T.J., Berenz A., Wicks J., Esquerra-Zwiers A., Sulo K.S., Gross M.E., Szotek J., Meier P., Patel A.L. (2020). The Economic Impact of Donor Milk in the Neonatal Intensive Care Unit. J. Pediatr..

[97] Weaver G., Bertino E., Gebauer C., Grovslien A., Mileusnic-Milenovic R., Arslanoglu S., Barnett D., Boquien C.Y., Buffin R., Gaya A. (2019). Recommendations for the establishment and operation of human milk banks in Europe: A consensus statement from the European Milk Bank Association (EMBA). Front. Pediatr..

[98] Vázquez-Román S., Bustos-Lozano G., López-Maestro M., Rodríguez-López J., Orbea-Gallardo C., Samaniego-Fernández M., Pallás-Alonso C.R. (2014). Clinical impact of opening a human milk bank in a neonatal unit. An. Pediatría.

[99] Moro G.E., Billeaud C., Rachel B., Calvo J., Cavallarin L., Christen L., Escuder-Vieco D., Gaya A., Lembo D., Wesolowska A. (2019). Processing of Donor Human Milk: Update and Recommendations From the European Milk Bank Association (EMBA). Front. Pediatr..

[100] Kontopodi E., Hettinga K., Stahl B., van Goudoever J.B., van Elburg R.M. (2022). Testing the effects of processing on donor human Milk: Analytical methods. Food Chem..

[101] Pitino M.A., Beggs M.R., O’Connor D.L., Doyen A., Pouliot Y., Sergius-Ronot M., Unger S. (2023). Donor human milk processing and its impact on infant digestion: A systematic scoping review of in vitro and in vivo studies. Adv. Nutr..

[102] Wong R.K., Pitino M.A., Mahmood R., Yihang Zhu I., Stone D., O’Connor D.L., Unger S., Chan T.C.Y. (2021). Predicting Protein and Fat Content in Human Donor Milk Using Machine Learning. J. Nutr..

[103] Peila C., Emmerik N.E., Giribaldi M., Stahl B., Ruitenberg J.E., van Elburg R.M., Moro G.E., Bertino E., Coscia A., Cavallarin L. (2017). Human Milk Processing. J. Pediatr. Gastroenterol. Nutr..

[104] Escuder-Vieco D., Espinosa-Martos I., Rodríguez J.M., Corzo N., Montilla A., Siegfried P., Pallás-Alonso C.R., Fernández L. (2018). High-Temperature Short-Time Pasteurization System for Donor Milk in a Human Milk Bank Setting. Front. Microbiol..

[105] Tran H., Nguyen T., Mathisen R. (2020). The use of human donor milk. BMJ.

[106] Zhang S., Li T., Xie J., Zhang D., Pi C., Zhou L., Yang W. (2021). Gold standard for nutrition: A review of human milk oligosaccharide and its effects on infant gut microbiota. Microb. Cell Fact..

[107] Ma J., Li Z., Zhang W., Zhang C., Zhang Y., Mei H., Zhuo N., Wang H., Wang L., Wu D. (2020). Comparison of gut microbiota in exclusively breast-fed and formula-fed babies: A study of 91 term infants. Sci. Rep..

[108] Liu Z., Roy N.C., Guo Y., Jia H., Ryan L., Samuelsson L., Thomas A., Plowman J., Clerens S., Day L. (2015). Human breast milk and infant formulas differentially modify the intestinal microbiota in human infants and host physiology in rats. J. Nutr..

[109] Moore R.E., Townsend S.D. (2019). Temporal development of the infant gut microbiome. Open Biol..

[110] Forbes J.D., Azad M.B., Vehling L., Tun H.M., Konya T.B., Guttman D.S., Field C.J., Lefebvre D., Sears M.R., Becker A.B. (2019). Association of exposure to formula in the hospital and subsequent infant feeding practices with gut microbiota and risk of overweight in the first year of life. JAMA Pediatr..

[111] Wang Z., Neupane A., Vo R., White J., Wang X., Marzano S.L. (2020). Comparing gut microbiome in mothers’ own breast milk- and formula-fed moderate-late preterm infants. Front. Microbiol..

[112] Ríos-Covián D., Ruas-Madiedo P., Margolles A., Gueimonde M., de Los Reyes-Gavilán C.G., Salazar N. (2016). Intestinal short chain fatty acids and their link with diet and human health. Front. Microbiol..

[113] Sisk P.M., Lambeth T.M., Rojas M.A., Lightbourne T., Barahona M., Anthony E., Auringer S.T. (2017). Necrotizing enterocolitis and growth in preterm infants fed predominantly maternal milk, pasteurized donor milk, or preterm formula: A retrospective study. Am. J. Perinatol..

[114] Zamrik S., Giachero F., Heldmann M., Hensel K.O., Wirth S., Jenke A.C. (2018). Impact of an in-house pediatric surgery unit and human milk centered enteral nutrition on necrotizing enterocolitis. BioMed Res. Int..

[115] Cristofalo E.A., Schanler R.J., Blanco C.L., Sullivan S., Trawoeger R., Kiechl-Kohlendorfer U., Dudell G., Rechtman D.J., Lee M.L., Lucas A. (2013). Randomized trial of exclusive human milk versus preterm formula diets in extremely premature infants. J. Pediatr..

[116] Hermann K., Carroll K. (2014). An exclusively human milk diet reduces necrotizing enterocolitis. Breastfeed. Med..

[117] Gopalakrishna K.P., Macadangdang B.R., Rogers M.B., Tometich J.T., Firek B.A., Baker R., Ji J., Burr A.H.P., Ma C., Good M. (2019). Maternal IgA protects against the development of necrotizing enterocolitis in preterm infants. Nat. Med..

[118] Wang L., Collins C., Ratliff M., Xie B., Wang Y. (2017). Breastfeeding reduces childhood obesity risks. Child. Obes..

[119] Lee J.W., Lee M., Lee J., Kim Y.J., Ha E., Kim H.S. (2019). The protective effect of exclusive breastfeeding on overweight/obesity in children with high birth weight. J. Korean Med. Sci..

[120] McCory C., Layte R. (2012). Breastfeeding and risk of overweight and obesity at nine-years of age. Soc. Sci. Med..

[121] Zhao Y.L., Ma R.M., Huang Y.K., Liang K., Ding Z.B. (2013). Effect of breastfeeding on childhood overweight in the offspring of mothers with gestational diabetes mellitus. Zhongguo Dang Dai Er Ke Za Zhi.

[122] Novaes J.F., Lamounier J.A., Colosimo E.A., Franceschini S.C., Priore S.E. (2012). Breastfeeding and obesity in Brazilian children. Eur. J. Public Health..

[123] Martin R.M., Patel R., Kramer M.S., Guthrie L., Vilchuck K., Bogdanovich N., Sergeichick N., Gusina N., Foo Y., Palmer T. (2013). Effects of promoting longer-term and exclusive breastfeeding on adiposity and insulin-like growth factor-I at age 11.5 years: A randomized trial. JAMA.

[124] Weber M., Grote V., Closa-Monasterolo R., Escribano J., Langhendries J.P., Dain E., Giovannini M., Verduci E., Gruszfeld D., Socha P. (2014). Lower protein content in infant formula reduces BMI and obesity risk at school age: Follow-up for a randomized trial. Am. J. Clin. Nutr..

[125] Escribano J., Luque V., Ferre N., Mendez-Riera G., Koletzko B., Grote V., Demmelmair H., Bluck L., Wright A., Closa-Monasterolo R. (2012). Effect of protein intake and weight gain velocity on body fat mass at 6 months of age: The EU Childhood Obesity Programme. Int. J. Obes..

[126] Salgin B., Norris S.A., Prentice P., Pettifor J.M., Richter L.M., Ong K.K., Dunger D.B. (2015). Even transient rapid infancy weight gain is associated with higher BMI in young adults and earlier menarche. Int. J. Obes..

[127] Klopp A., Vehling L., Becker A.B., Subbarao P., Mandhane P.J., Turvey S.E., Lefebvre D.L., Sears M.R., Azad M.B., CHILD Study Investigators (2019). Modes of infant feeding and the risk of childhood asthma: A prospective birth cohort study. J. Pediatr..

[128] Chu S., Chen Q., Chen Y., Bao Y., Wu M., Zhang J. (2017). Cesarean section without medical indication and risk of childhood asthma, and attenuation by breastfeeding. PLoS ONE.

[129] Elbert N., van Meel E.R., den Dekker H.T., de Jong N.W., Nijsten T.E.C., Jaddoe V.W.V., de Jongste J.C., Pasmans S.G.M.A., Duijts L. (2017). Duration and exclusiveness of breastfeeding and risk of childhood atopic diseases. Allergy.

[130] Greer F.R., Sicherer S.H., Burks A.W. (2019). The effects of early nutritional interventions on the development of atopic disease in infants and children: The role of maternal dietary restriction, breastfeeding, hydrolized formulas, and timing of introduction of allergenic complementary foods. Pediatrics.

[131] Lee S.A., Lim J.Y., Kim B.S., Cho S.J., Kim N.Y., Kim O.B., Kim Y. (2015). Comparison of the gut microbiota profile in breast-fed and formula-fed Korean infants using pyrosequencing. Nutr. Res. Pract..

[132] Praveen P., Jordan F., Priami C., Morine M.J. (2015). The role of breast-feeding in infant immune-system: A systems perspective on the intestinal microbiome. Microbiome.

[133] Rodriguez-Herrera A., Mulder K., Bouritius H., Rubio R., Muñoz A., Agosti M., Lista G., Corvaglia L., Ludwig T., Abrahamse-Berkeveld M. (2019). Gastrointestinal tolerance, growth and safety or a partly fermented formula with specific prebiotics in healthy infants: A double-blind, randomized controlled trial. Nutrients.

[134] Haaman M., Knol J. (2005). Quantitative real-time PCR assays to identify and quantify fecal Bifidobacterium species in infants receiving a prebiotic infant formula. Appl. Environ. Microbiol..

[135] Salvini F.J., Riva E., Salvatici E., Boehm G., Jelinek J., Banderali G., Giovannini M. (2011). A specific probiotic mixture added to starting infant formula has long-lasting bifidogenic effects. J. Nutr..

[136] Ballard O., Morrow A.L. (2013). Human milk composition: Nutrients and bioactive factors. Pediatr. Clin. N. Am..

[137] Widger J., O’Connell N.H., Stack T. (2010). Breast milk causing neonatal sepsis and death. Clin. Microbiol. Infect..

[138] Sullivan S., Schanler R.J., Kim J.H., Patel A.L., Trawöger R., Kiechl-Kohlendorfer U., Chan G.M., Blanco C.L., Abrams S., Cotton C.M. (2010). An exclusively human milk-based diet is associated with a lower rate of necrotizing enterocolitis than a diet of human milk and bovine milk-based products. J. Pediatr..

[139] Gnawali A. (2021). Prematurity and the risk of development of childhood obesity: Piecing together the pathophysiological puzzle. A literature review. Cureus.

[140] Zhang Y., Deng Q., Wang J., Wang H., Li Q., Zhu B., Ji C., Xu X., Johnston L. (2022). The impact of breast milk feeding on early brain development in preterm infants in China: An observational study. PLoS ONE.

[141] Stinson L.F., Ma J., Lai C.T., Rea A., Perrella S.L., Geddes D.T. (2024). Milk microbiome transplantation: Recolonizing donor milk with mother’s own milk microbiota. Appl. Microbiol. Biotechnol..

[142] Cacho N.T., Harrison N.A., Parker L.A., Padgett K.A., Lemas D.J., Marcial G.E., Li N., Carr L.E., Neu J., Lorca G.L. (2017). Personalization of the Microbiota of Donor Human Milk with Mother’s Own Milk. Front. Microbiol..

[143] Mallardi D., Tabasso C., Piemontese P., Morandi S., Silvetti T., Biscarini F., Cremonesi P., Castiglioni B., Pica V., Stuknyte M. (2021). Inoculation of mother’s own milk could personalize pasteurized donor human milk used for feeding preterm infants. J. Transl. Med..

[144] Torrez Lamberti M.F., Harrison N.A., Bendixen M.M., DeBose-Scarlett E.M., Thompson S.C., Neu J., Parker L.A., Lorca G.L. (2021). Frozen Mother’s Own Milk Can Be Used Effectively to Personalize Donor Human Milk. Front. Microbiol..

[145] Kang W., Pan L., Peng C., Dong L., Cao S., Cheng H., Wang Y., Zhang C., Gu R., Wang J. (2020). Isolation and characterization of lactic acid bacteria from human milk. J. Dairy Sci..

[146] Liu W., Chen M., Duo L., Wang J., Guo S., Sun H., Menghe B., Zhang H. (2020). Characterization of potentially probiotic lactic acid bacteria and bifidobacteria isolated from human colostrum. J. Dairy Sci..

[147] Zhang S., Chen L., Hu M., Zhu J. (2023). 2′-Fucosyllactose (2′-FL) changes infants gut microbiota composition and their metabolism in a host-free human colonic model. Food Res. Int..

[148] Salli K., Hirvonen J., Siitonen J., Ahonen I., Anglenius H., Maukonen J. (2021). Selective Utilization of the Human Milk Oligosaccharides 2′-Fucosyllactose, 3-Fucosyllactose, and Difucosyllactose by Various Probiotic and Pathogenic Bacteria. J. Agric. Food Chem..

[149] Nogacka A.M., Arboleya S., Nikpoor N., Auger J., Salazar N., Cuesta I., Mantecón L., Solís G., Gueimonde M., Tompkins T.A. (2021). Influence of 2′-Fucosyllactose on the Microbiota Composition and Metabolic Activity of Fecal Cultures from Breastfed and Formula-Fed Infants at Two Months of Age. Microorganisms.

[150] Zabel B.E., Gerdes S., Evans K.C., Nedveck D., Koch Singles S., Volk B., Budinoff C. (2020). Strain-specific strategies of 2′-fucosyllactose, 3-fucosyllactose, and difucosyllactose assimilation by *Bifidobacterium longum* subsp. infantis Bi-26 and ATCC 15697. Sci. Rep..

[151] Laursen M.F., Pekmez C.T., Wange Larsson M., Vendelbo Lind M., Yonemitsu C., Larnkjær A., Mølgaard C., Bode L., Ove Dragsted L., Michaelsen K.F. (2021). Maternal milk microbiota and oligosaccharides contribute to the infant gut microbiota assembly. ISME Commun..

